# Grouped immunogens are hierarchically recognized by host to generate antibodies

**DOI:** 10.3389/fimmu.2026.1778237

**Published:** 2026-04-17

**Authors:** Xiang Liu, Xuanxian Peng, Hui Li, Xianjie Liu

**Affiliations:** 1 State Key Laboratory of Bio-Control, School of Life Sciences, Southern Marine Science and Engineering Guangdong Laboratory (Zhuhai), Guangdong Key Laboratory of Pharmaceutical Functional Genes, Sun Yat-sen University, Guangzhou, China; 2 Anhui Province Key Laboratory of Embryo Development and Reproductive Regulation, Anhui Province Key Laboratory of Environmental Hormone and Reproduction, School of Biological and Food Engineering, Fuyang Normal University, Fuyang, China; 3 Shenzhen International Institute for Biomedical Research, Shenzhen, China

**Keywords:** antibody, B cell, hierarchy, host, immunogens, metabolic process, transport

## Abstract

**Introduction:**

Microorganisms with dozens, hundreds, or thousands of proteins invade hosts. Whether the hosts simultaneously recognize all or a part of them is unknown.

**Methods:**

A total of 68 *Escherichia coli* recombinant outer membrane (OM) proteins were pooled to immunize mice, and the resulting antisera were analyzed using a protein microarray. Proteins recognized in each cycle were removed, and the remaining proteins were re-pooled for subsequent immunizations. Bioinformatics analysis of B cell epitope scores and GO functional categories was performed across the identified immunogen hierarchies.

**Results:**

Among the 68 OM proteins, only 18 were recognized to generate antibodies, designated as the first hierarchy of immunogens. When the other 50 proteins were grouped to immunize mice, 15 were detected to have their corresponding antibodies, designated as the second hierarchy of immunogens. This procedure was repeated four times, leading to the identification of 16 and eight proteins as the third and the fourth hierarchy of immunogens, respectively, and 11 residual proteins as the fifth hierarchy of immunogens. Bioinformatics analysis showed a negative correlation of B cell epitope score with increasing hierarchies, suggesting that the score plays an essential role in hierarchy recognition. In addition, more proteins with transport and fewer proteins with metabolic process were found in the combination of the first, second, and third than the combination of the fourth and fifth hierarchies of immunogens.

**Discussion:**

The host's immune system hierarchically recognizes antigens to mount antibody responses. This finding highlights the way in understanding differentially neutralizing antibodies during infections caused by different microorganisms.

## Introduction

Microbial pathogens infect hosts, and the hosts mount an immune response including antibodies against the pathogens (1, 2). However, not all antibodies can directly eliminate pathogens (3, 4). This is because antibodies are classified into neutralizing and non-neutralizing antibodies. Only neutralizing antibodies play a particularly critical role because they directly block pathogen entry into host cells and are often strongly associated with protective immunity (5, 6). The neutralizing and non-neutralizing antibodies are generated from a host’s immune response to neutralizing and non-neutralizing immunogens, respectively (7–9). Therefore, whether the neutralizing immunogens are recognized by hosts to generate neutralizing antibodies is especially key for implementing anti-infective immunity.

Bacteria and viruses consist of thousands of and hundreds of proteins (10, 11), respectively, where neutralizing immunogens are included (12). All proteins of these microbes can be recognized as immunogens by the host’s immune system to generate antibodies when each of these proteins separately challenges the host. However, it seems that not all antibodies targeting at the thousands and hundreds of proteins can be simultaneously generated during the bacterial and viral infections, respectively, although information regarding how many antibodies to these proteins are generated is not available. This is supported by a recent report that out of 69 *Escherichia coli* outer membrane (OM) proteins, an average of 22%–29% corresponding antibodies are detected among individuals of different ages, with no and few antibodies to about half of these OM proteins (13). This suggests that only a part of the bacterial proteins is recognized by the host in a bacterial infection. This poses a question on whether biological regulation or physical barrier causes the failure of the immune response to all proteins. Logically, bacterial components meted by hosts include the most outside proteins of the cells, secretory proteins, and all proteins from destroyed cells by the host’s immune system, suggesting their importance in biological regulation. This can be answered by a pool with single proteins to challenge the host since the pooled proteins are free in physical barrier.

Bacterial outer membrane (OM) proteins consist of dozens of proteins and are located in the outermost part of the cells (14). These OM proteins are firstly recognized by hosts and thus can be regarded as an ideal pool to identify vaccine candidates (15, 16). Therefore, OM proteins are an ideal model to explore how the host recognizes the dozens of OM proteins and thus understand whether all proteins are simultaneously recognized by the host for antibodies to be generated.

In this study, 68 *Escherichia coli* recombinant OM proteins were pooled to immunize mice to know whether all or only a part of these OM proteins stimulate antibody production. If only a part of them caused an immune response, these stimulated OM proteins were removed from the pool with the 68 OM proteins, and the other OM proteins were used to construct the next pool to immunize the mice with. This process was repeated four times. The first, second, third, and fourth cycled OM proteins and the remaining OM proteins were designated as the first, second, third, fourth, and fifth hierarchies of immunogens. This study showed that the pooled OM proteins are hierarchically recognized by the host immune system for it to mount antibody responses.

## Materials and methods

### OM proteins, bacterial strains, and animals

A total of 68 purified OM proteins of *E. coli* were the same as in the previous report (13). Enteropathogenic *E. coli* Y-17 was preserved in our laboratory. Kunming mice weighing 20 ± 1 g were purchased from Sun Yat-sen University. All animal work was conducted in strict accordance with the recommendations of the Guide for the Care and Use of Laboratory Animals of the National Institutes of Health. The protocol was approved by the Institutional Animal Care and Use Committee of Sun Yat-sen University (approval no. SYSU-IACUC-2020-B126716).

### Extraction of *E. coli* OM proteins for affinity chromatography of antisera

The extraction was carried out as described previously (16). Single colonies of *E. coli* BW25113 were selected and inoculated into 5 mL of LB medium overnight. The bacterial culture was transferred into 500 mL of fresh LB medium at a volume ratio of 1:100 and incubated at 37°C at 200 rpm/min until OD_600_ = 1.0. After centrifuging at 8,000 rpm/min for 2 min, the bacterial cells were suspended in 10 mL of 50 mM Tris-HCl (pH 7.4) buffer and sonicated for 40 min in ice water. The sonicated solution was centrifuged at 5,000 *g* at 4°C for 20 min. The supernatants were collected and centrifuged at 4°C for 1 h at 100,000 *g* to enrich the OM proteins. The enriched OM proteins were dissolved in 50 mM Tris-HCl (pH 7.4) for subsequent use. The mouse serum used as a control tested negative for the mixed OMP antibodies, confirming the absence of pre-existing immunity.

### Preparation of mouse antisera against different hierarchies of immunogens of OM proteins

Each 3 µg (vector was deducted) of the first, second, third, and fourth hierarchies of 68, 50, 33, and 17 purified OM proteins of *E. coli*, respectively, were pooled in 50 mM Tris-HCl (pH 7.4) buffer to construct the four pooled groups of immunogens. Molar equivalents were not calculated due to the differing molecular weights of the antigens. These immunogens were emulsified with Freund’s complete adjuvant or with Freund’s incomplete adjuvant and utilized as the primary and boost immunogens. The boost immunization was performed at 14 days after the primary immunization. Antisera were collected at 7 days after the boost immunization. An equal volume of antisera from five mice was pooled. These pooled antisera were used as the primary antibody in protein microarray detection.

### Purification of mouse antisera against OM proteins by affinity chromatography

*E. coli* OM proteins at 300 µg were isolated by using SDS-PAGE electrophoresis and then transferred into a nitrocellulose (NC) membrane at 80 V at 4°C for 1 h. Then, the NC membrane was cut into a size of 2 mm^2^, mixed with 500 µL of 1:5 dilutions of antisera, and incubated at 4°C overnight. After the NC membrane was washed four times with PBS buffer, 500 µL of gly-HCl (pH 2.4) buffer was added at room temperature for 10 min; then, it was adjusted to pH 7.0 with 40 µL of 1.5 M Tris (pH 8.8) solution and stored in a refrigerator at -20 °C for later use.

### Preparation of protein microarray to detect the antiserum of OM proteins

The preparation of protein microarray was carried out as described previously (13). In brief, NC membrane was divided into uniform squares of 0.7 × 0.7 cm and soaked with water. The purified *E. coli* recombinant OM proteins (2 µL containing 0.5 µg) were spotted in the middle of the squares on the NC membrane to prepare the OM protein microarray. The protein microarray was placed in 5% milk TNT solution to block at room temperature for 2 h. The purified antisera against OM proteins were incubated with the microarray at 37°C for 40 min. After washing three times with TNT buffer, rabbit anti-mouse secondary antibody (Sigma, St. Louis, USA) was added to the chip, and the mixture was incubated at 37°C for 1 h. Finally, DAB method was used to visualize points in the protein microarray.

### Detection of the immunoprotective effects of OM proteins

Detection was carried out as described previously. The immune protection assays for 22 OM proteins were performed as described previously (13). The resulting data are re-analyzed in this study to compare the protective efficacy across the five immunogen hierarchies.

### Prediction of the physical, chemical, and immunological characteristics of OM proteins

The prediction was performed using an online website—specifically, for B cell epitope: https://services.healthtech.dtu.dk/services/BepiPred-2.0/and for T cell epitope: http://tools.iedb.org/mhci/. The GO function of OM proteins used data from the SWISS-PROT protein database. The others were processed using https://www.expasy.org/.

## Results

### Hierarchy of OM immunogens is identified based on antibody generation

**Figure 1:**
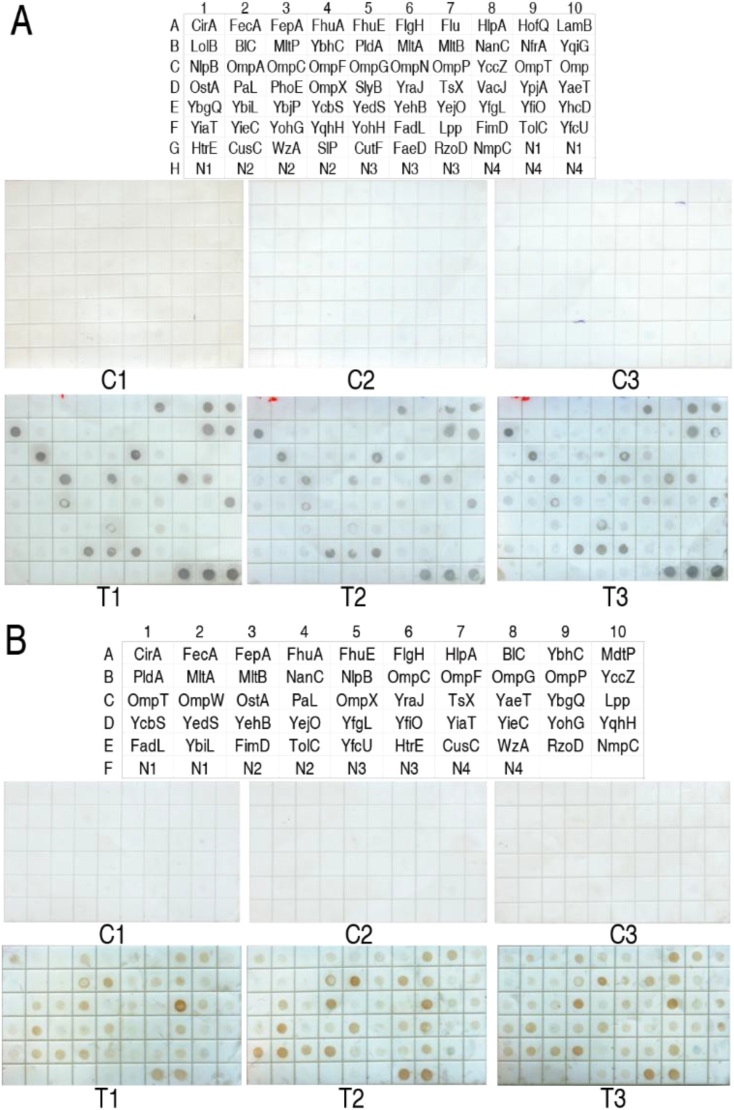
Immune reaction between recombinant OM proteins on protein arrays and antibodies purified from NC membrane coated with natural OM proteins for the identification of the first and second hierarchies of immunogens. **(A)** Identification of the first hierarchy of immunogens. Location of 68 OM proteins and the immune reaction. The stained spots are the first hierarchy of immunogens. N1, 50 mM Tris-HCl; N2, BSA, bovine serum albumin; N3, pET-32 fusion protein. The three were used as negative controls. N4, 0.5 µg supernatant of *E. coli* BW25113 lysate solution as a positive control. **(B)** Identification of the second hierarchy of immunogens. Location of 50 OM proteins and the immune reaction. The stained spots are the second hierarchy of immunogens. N1, 50 mM Tris-HCl; N2, BSA; N3, pET-32 fusion protein. The three were used as negative controls. N4, 0.5 µg supernatant of *E. coli* BW25113 lysate solution as a positive control. C1, C2, and C3 are control 1, control 2, and control 3, indicating that mouse pre-immune serum was used as the primary antibody. T1, T2, and T3 are the tested groups, indicating that mouse immune serum was used as the primary antibody.

**Figure 2:**
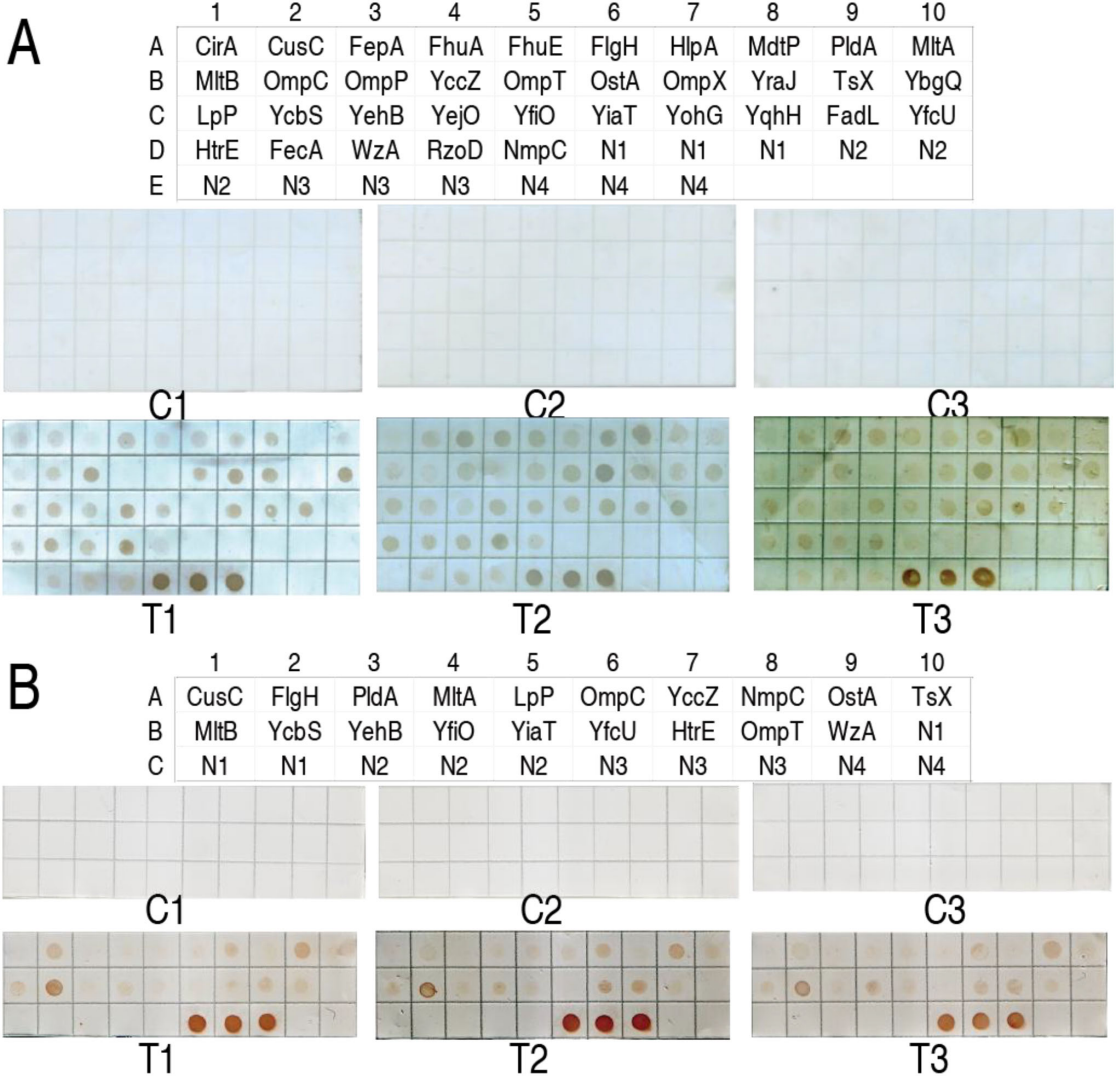
Immune reaction between recombinant OM proteins on protein arrays and antibodies purified from NC membrane coated with natural OM proteins for identification of the third and fourth hierarchies of immunogens. **(A)** Identification of the third hierarchy of immunogens. Location of 35 OM proteins and the immune reaction. The stained spots are the third hierarchy of immunogens. N1, 50 mM Tris-HCl; N2, BSA; N3, pET-32 fusion protein. The three were used as negative controls. N4, 0.5 µg supernatant of *E. coli* BW25113 lysate solution as a positive control. **(B)** Identification of the fourth hierarchy of immunogens. Location of 19 OM proteins and the immune reaction. The stained spots are the fourth hierarchy of immunogens. N1, BSA (negative control); N2, pET-32a fusion protein (negative control); N3, 0.5 µg supernatant of *E. coli* BW25113 lysate solution (positive control); N4, 50 mM Tris-HCl (negative control). C1, C2, and C3 are control 1, control 2, and control 3, indicating that mouse pre-immune serum was used as the primary antibody. T1, T2, and T3 are the tested groups, indicating that mouse immune serum was used as the primary antibody.

To know which hierarchy recognized by the host’s immune system the OM proteins belong to, 68 *E. coli* recombinant OM proteins at 3 μg each were pooled to immunize mice. The resulting antisera from five mice were pooled and reacted with a protein array with the 68 recombinant OM proteins, where the primary antibodies were purified by nitrocellulose membrane coated with natural *E. coli* OM proteins. Among the 68 OM proteins, 18 exhibited a reaction with the purified antibodies, suggesting that they are recognized by the host’s immune system in the first cycle of immunization and designated as the first hierarchy of immunogens. The first hierarchy of immunogens consisted of Flu, HofQ, LamB, LolB, NfrA, YqiG, OmpA, OmpN, PhoE, SlyB, VacJ, YpjA, YbjP, YhcD, YohH, SlP, CutF, and FaeD (**Figure 1A**). Then, the 18 immunogens were removed from the pool with 68 OM proteins. The residue stock of the other 50 OM proteins was used to immunize mice. Among them, 15 OM proteins were recognized as the second hierarchy of OM immunogens. They were BlC, YbhC, NanC, BamC, OmpF, OmpG, OmpW, PaL, BamA, YedS, BamB, YieC, YbiL, FimD, and TolC (**Figure 1B**). The same procedure identified 16 (CirA, FepA, FhuA, FhuE, HlpA, MdtP, OmpP, OmpX, YraJ, YbgQ, Yejo, YohG, YqhH, FadL, FecA, and RzoD) and eight (FlgH, YccZ, OstA, MltB, YcbS, BamD, HtrE, and OmpT) OM proteins as the third and fourth hierarchies of immunogens, respectively (**Figures 2A, B**). Finally, the residue stock of the other 11 OM proteins (CusC, PldA, MltA, LpP, OmpC, NmpC, TsX, YehB, YiaT, YfcU, and WzA) was called as the fifth hierarchy of immunogens. These results indicate that the host’s immune system hierarchically recognizes antigens to mount antibody responses.

### Immune protection of different hierarchies of OM proteins

To know the relationship between immune protection and the different hierarchies of immunogens, 22 of the 68 recombinant OM proteins were randomly selected to separately immunize mice. The protective efficacy of individual OM proteins was described previously (13). These mice were challenged with a virulent *E. coli* Y17 (17). Specifically, among the 22 OM proteins, three (OmpA, VacJ, and OmpN), four (OmpW, TolC, NanC, and BIC), four (HIpA, MdtP, FepA, and OmpX), two (OstA and TsX), and four (OmpC, YfcU, PldA, WzA, and YiaT) belonged to the first, second, third, fourth, and fifth hierarchies of immunogens, respectively. Significant immune protection was detected in mice immunized with OmpA (54.54%), BamC (63.64%), HlpA (63.64%), OstA (81.82%), TsX (63.64%), OmpC (63.64%), and YfcU (63.64%). They belonged to the first (OmpA), second (BamC), third (HlpA), fourth (OstA and TsX), and fifth (OmpC and YfcU) hierarchies of immunogens (13). These results indicate that the immune protection is not related to the hierarchy of immunogens.

### Physical, chemical, and immunological characteristics across different hierarchies of immunogens

**Figure 3:**
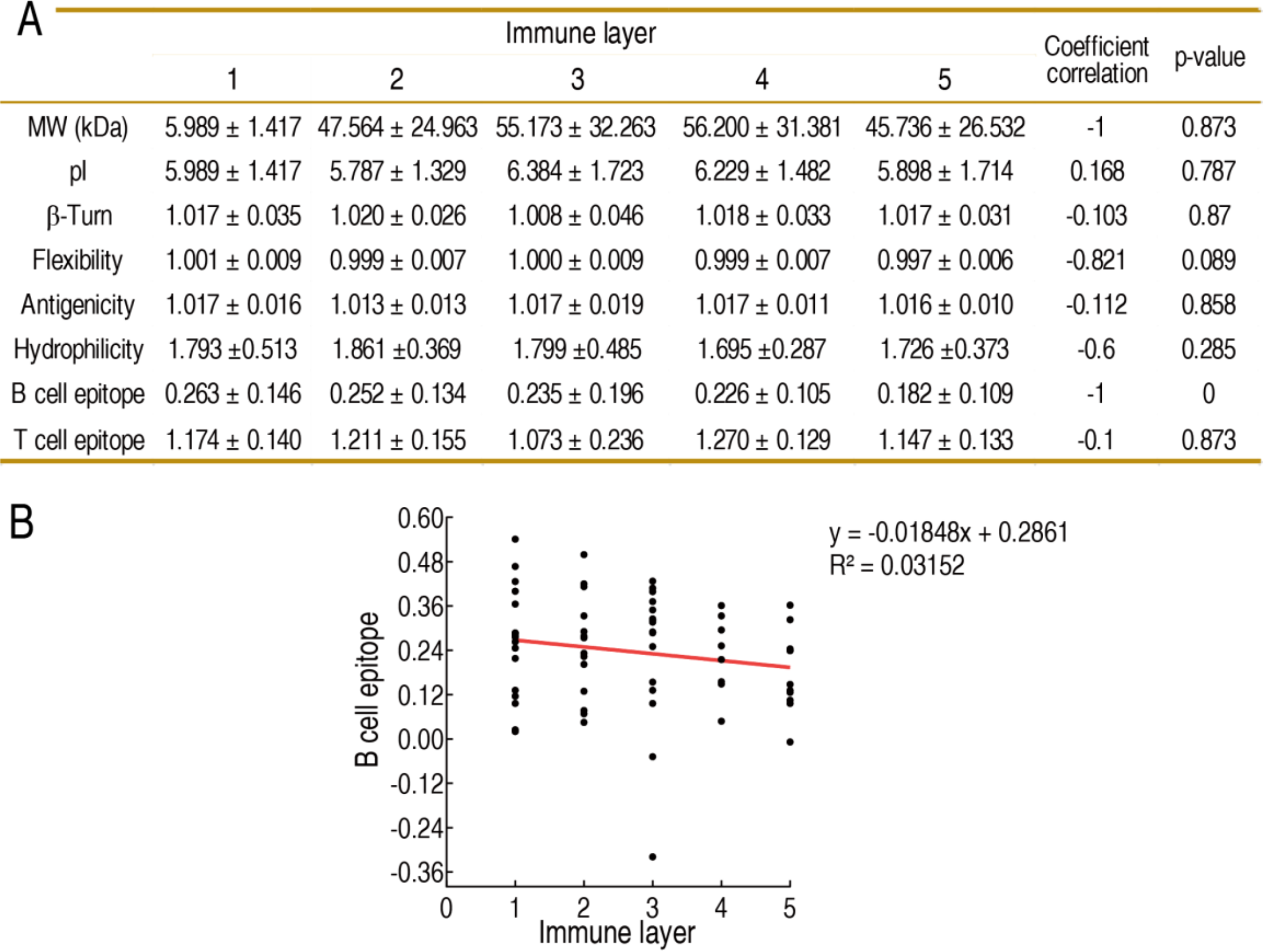
Relationship between physical, chemical, and immunological characteristics and different cycled OM proteins. **(A)** Physical, chemical, and immunological characteristics in different cycled OM proteins. Values are presented as mean ± standard deviation (SD). **(B)** Correlation between B cell epitope and different cycled OM proteins. The red dotted line indicates the fitted linear regression line, showing the negative correlation between B cell epitope score and immunogen hierarchy. Linear regression analysis was performed using GraphPad Prism 8 software to determine the trend of B cell epitope values for the five immune layer proteins, along with the linear equation and the goodness of fit (*R*^2^).

To explore which physical, chemical, and immunological characteristics are related to the hierarchy of immunogens, molecular mass, isoelectric point, β-Turn, flexibility, antigenicity, hydrophilicity, B cell epitope, and T cell epitope were analyzed in different hierarchies of immunogens. There were no differences between molecular mass, isoelectric point, β-Turn, flexibility, antigenicity, hydrophilicity, T cell epitope, and the different hierarchies of immunogens (**Figure 3A**; **Supplementary Tables S1**–**S5**). However, 0.263 ± 0.146, 0.252 ± 0.134, 0.235 ± 0.196, 0.226 ± 0.105, and 0.182 ± 0.109 B cell epitopes were annotated in the first, second, third, fourth, and fifth hierarchies of immunogens. The correlation analysis showed that the epitope scores were negatively correlated to the hierarchy of immunogens, suggesting that B cell epitope reduced with increasing hierarchy of immunogens (**Figures 3A, B**; **Supplementary Tables S1**–**S5**).

### GO function across different hierarchies of immunogens

**Figure 4:**
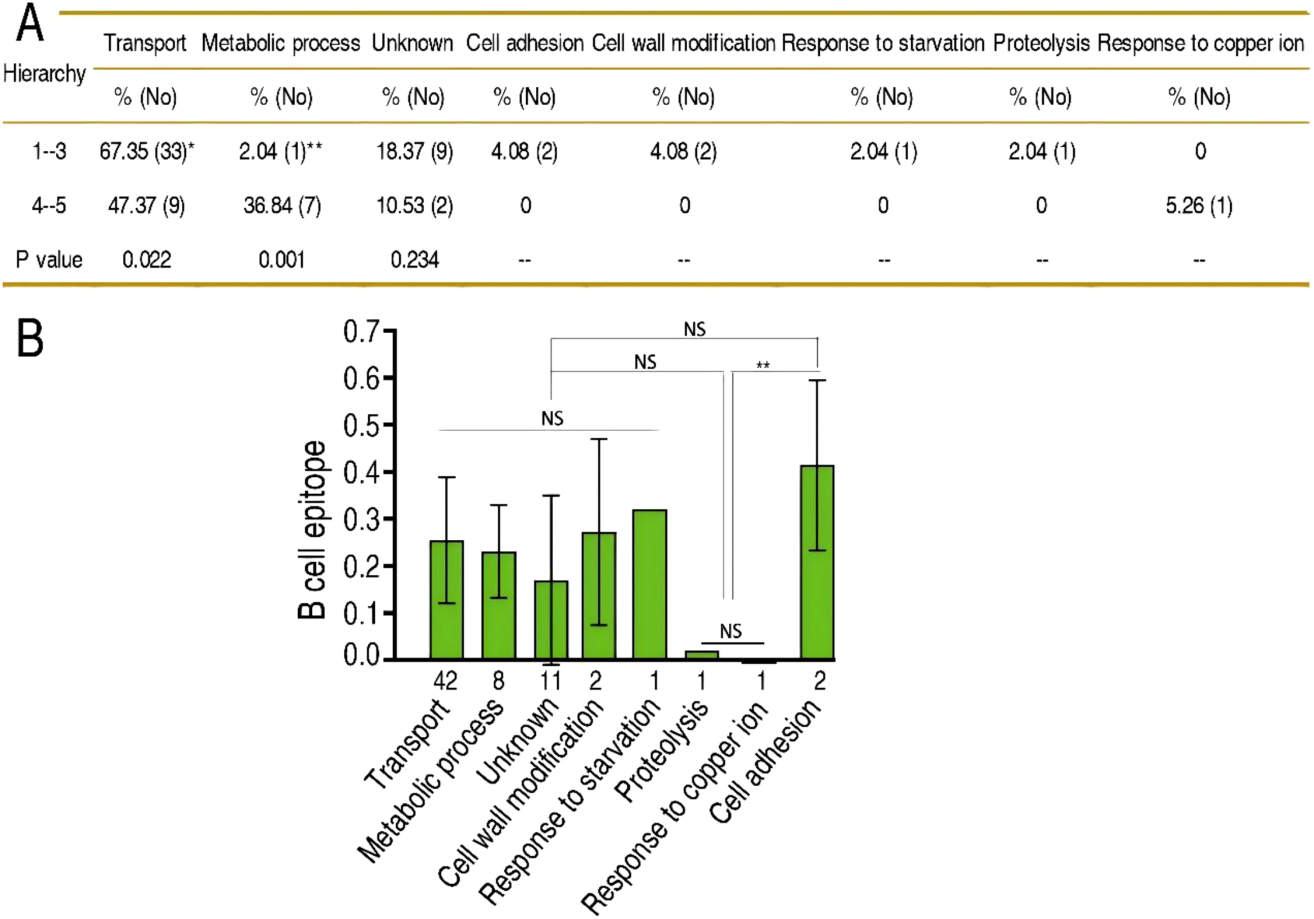
GO function and immunogen hierarchy. **(A)** Difference in GO function between the combination of the first, second, and third hierarchies and the combination of the fourth and fifth hierarchies. **(B)** Average of the B cell epitope values of proteins with different functions in the GO classification. The horizontal axis represents the number of proteins with the same function in the GO classification, while the vertical axis shows the average B cell epitope values of proteins. Differences between groups were analyzed using one-way ANOVA with Tukey’s s-b (k) *post-hoc* test in SPSS software. *p < 0.05, **p < 0.01; NS, not significant (*p* > 0.05).

GO analysis was performed to categorize the function of OM proteins and then to explore the relationship between the function and different hierarchies of immunogens. The five hierarchies of immunogens were integrated into two groups, namely: (1) the combination of the first, second, and third hierarchies and (2) the combination of the fourth and fifth hierarchies. Among the eight categories of function, transport and metabolic process exhibited differences between the two groups, but the others did not. Specifically, the percentages of OM proteins with transport function were 67.35% in group 1 and 47.37% in group 2, whereas those with metabolic process were 2.04% in group 1 and 36.84% in group 2 (**Figure 4A**). To further understand whether this functional bias is attributed to differential B cell epitope scores between transport OM proteins and metabolic process OM proteins, we analyzed the association of B cell epitope score with these functions. There were no differences between them in every function (**Figure 4B**). Therefore, OM proteins serving for transport and metabolic process are easier and harder to be recognized by the host immune system independently of their B cell epitope scores.

## Discussion

To explore whether a group of antigens simultaneously stimulates a host’s immune system to generate antibody response to every antigen, the present study pools 68 *E. coli* OM proteins to immunize mice and detect antibodies to the 68 OM proteins using a protein microarray. Interestingly, the mice hierarchically mount antibody response to the group of 68 OM proteins. Specifically, among the 68 OM proteins, 18, 15, 16, and eight are recognized by the first, second, third, and fourth cycles, respectively, and 11 (16.2%) are not recognized to generate antibodies after the four cycled immunizations. The 11 OM proteins consist of CusC, PldA, MltA, Lpp, OmpC, NmpC, TsX, YehB, YiaT, YfcU, and WzA. Among them, Lpp, OmpC, and TsX have been reported to be immunogenic, as specific antibodies against each of them have been successfully generated (18–20). These results indicate that the host cannot simultaneously mount an antibody response to a group of immunogens, suggesting the selectivity of immune antibody response. This interesting finding can be used to understand why non-neutralizing or weak-neutralizing antibody was found in infections caused by microbes, such as HCV and HIV infection, even if powerful neutralizing immunogens exist (21–24). Therefore, how to make the powerful neutralizing immunogens expose to the host immune system is especially key in developing specific antibody protection against bacterial and viral infections.

The present study shows that the hierarchy of immunogens is mostly dependent upon the B cell epitope. The B cell epitope is the specific portion of antigen that is recognized by the B cell receptor, which is able to elicit an antibody response (25). The identification of B cell epitopes plays a crucial role in the development of effective peptide vaccines (26, 27). The finding of a positive correlation between immunogen hierarchies and B cell epitopes score is consistent with the event that antibody generation is based upon the interaction between the B cell epitope and the B cell receptor (28, 29). Therefore, easier recognition and interaction by B cells play an essential role in categorizing the hierarchy of immunogens.

Comparatively, similar amounts of OM proteins are identified in the first, second, and third cycles, whereas only half or less than half of OM proteins are identified in the fourth cycles. This suggests possibly differential recognition among them. Thus, the first, second, and third hierarchies of immunogens and the fourth and fifth hierarchies of immunogens are merged into group 1 and group 2, respectively. More transport proteins are detected in group 1, while more proteins with metabolic process are found in group 2. There are significant differences between them. To exclude the possibility that these differences are related to the B cell epitope, the B cell epitope score is compared between every function of these proteins, including transport and metabolic process. No difference is detected between them. Therefore, the OM proteins with transport are easier to be recognized by the host’s immune system than the OM proteins with metabolic process.

The comparison of the hierarchical pattern identified in mice with our previous observations of human serum antibody responses to the same panel of *E. coli* OM proteins is informative. In that study (13), antibodies against all 69 OM proteins were detectable in human sera, but only a subset of proteins, such as OmpA, OmpX, TsX, HlpA, and FepA, elicited very high seroprevalence close to 100%, whereas many OM proteins induced low or undetectable antibody frequencies. Consistent with this, several OM proteins that we classify here as early hierarchies in mice overlap with those showing high seroprevalence and strong protective capacity in humans (for example, OmpA, BamC, and HlpA), while OM proteins that remain poorly immunogenic in mice after repeated immunization tend to correspond to antigens that elicit low seroprevalence in human sera. At the same time, there are notable exceptions—for instance, OmpC and YfcU are placed in the fifth hierarchy in the present mouse study but are frequently recognized and protective in humans—which may reflect repeated environmental exposure to *E. coli* or species−specific differences in antigen presentation and immune regulation. Taken together, these observations suggest that certain aspects of the hierarchical recognition pattern may be conserved across species while also highlighting the importance of integrating animal model data with human serology to understand the immune recognition of bacterial OM antigens.

It is important to note that in our immunization regimen, all antigens were administered at equal mass proportion (3 µg per protein), not at equimolar concentrations. Since the molecular weights of the tested proteins differ, this means that the molar quantity of each antigen presented to the immune system was not identical. This is a potential caveat when comparing the relative immunogenicity (the “hierarchy”) of these proteins, as smaller proteins may have been present at higher molar concentrations. However, given that the protective efficacy did not correlate with this hierarchy and the observed immunogenicity differences were substantial, we are confident that the core findings of this study remain valid. Future studies aiming to precisely rank intrinsic immunogenicity should employ equimolar antigen doses. Nevertheless, even equimolar dosing is an imperfect model; we therefore emphasize the qualitative nature of our findings.

A key limitation of our study is that the proteins were pooled based on equal mass proportion, whereas the hierarchy of immune recognition and response depends on molecular quantity. Even uniform molarity would not fully replicate physiological conditions, where antigen abundance, accessibility, and temporal expression vary dynamically during infections. Thus, our study focuses on qualitative differences in immunogenicity hierarchies based on protein identity (which antigens are recognized) rather than quantitative dose–response relationships. The consistent hierarchical patterns observed across repeated immunizations support the existence of intrinsic immunogenicity hierarchies, independent of absolute antigen quantity.

## Conclusion

The present study tries to explore how a host recognizes dozens of proteins all at once to generate antibodies. Our results show that the host cannot simultaneously recognize all of the dozens of proteins to produce antibodies. It hierarchically recognizes the grouped proteins, which is related to B cell epitope and OM protein function.

## Data Availability

The original contributions presented in the study are included in the article/**Supplementary Material**. Further inquiries can be directed to the corresponding author.
