# Supplementary material for: Grouped immunogens are hierarchically recognized by host to generate antibodies
Source: Front Immunol. 2026 Apr 17;17:1778237. doi: 10.3389/fimmu.2026.1778237 (PMC13132694; doi:10.3389/fimmu.2026.1778237)
Supplement: Supplementary file 1 [file Table1.docx]

Supplementary Tables

**Table S1 Physical, chemical, and immunological characteristics of the first hierarchy of immunogens**

| Protein | MW (kDa) | pI | β-Turn | Flexibility | Antigenicity | Hydrophilicity | B cell epitope | T cell epitope | Function |
| --- | --- | --- | --- | --- | --- | --- | --- | --- | --- |
| Flu | 107 | 5.78 | 1.041 | 1.013 | 1.005 | 2.428 | 0.467 | 1.2098 | Transport |
| HofQ | 44.7 | 5.97 | 0.934 | 1 | 1.04 | 1.349 | 0.025 | 1.1572 | Transport |
| LamB | 49.9 | 4.81 | 1.031 | 0.997 | 0.994 | 1.891 | 0.276 | 1.2108 | Transport |
| LolB | 21 | 8.89 | 1.024 | 1.011 | 1.012 | 1.69 | 0.287 | 1.2928 | Transport |
| NfrA | 111 | 6.52 | 0.969 | 0.993 | 1.024 | 1.459 | 0.116 | 1.391 | Unknown |
| YqiG | 90 | 5.16 | 1.042 | 1.002 | 1.011 | 1.86 | 0.263 | 1.1996 | Transport |
| OmpA | 37.2 | 5.99 | 1.02 | 0.999 | 1.021 | 1.895 | 0.4 | 1.1306 | Transport |
| OmpN | 41.2 | 4.47 | 1.056 | 0.997 | 0.994 | 2.275 | 0.365 | 1.0566 | Transport |
| PhoE | 38.9 | 4.93 | 1.033 | 1.002 | 0.994 | 2.201 | 0.286 | 1.2234 | Transport |
| SlyB | 15.6 | 9.36 | 1.018 | 1.006 | 1.036 | 2.124 | 0.281 | 0.9998 | Unknown |
| VacJ | 27 | 4.86 | 1.022 | 0.994 | 1.019 | 1.164 | 0.132 | 1.2818 | Unknown |
| YpjA | 157.5 | 5.18 | 1.054 | 1.02 | 0.999 | 2.603 | 0.541 | 1.3382 | Cell adhesion |
| YbjP | 18.99 | 6.05 | 1.024 | 1.004 | 1.029 | 1.874 | 0.218 | 1.1896 | Unknown |
| YhcD | 86.3 | 4.8 | 1.032 | 1.006 | 1.015 | 1.915 | 0.284 | 1.2338 | Transport |
| YohH | 10.4 | 7.78 | 0.96 | 0.981 | 1.046 | 0.726 | 0.096 | 0.8454 | Transport |
| SlP | 20.9 | 6.82 | 1.004 | 0.993 | 1.038 | 0.868 | 0.02 | 1.0496 | Response to starvation |
| CutF | 25.8 | 5 | 0.986 | 0.999 | 1.013 | 1.788 | 0.246 | 0.999 | Xenobiotic metabolic process |
| FaeD | 85 | 5.43 | 1.06 | 1.009 | 1.016 | 2.172 | 0.426 | 1.3232 | Transport |

**Table S2 Physical, chemical, and immunological characteristics of the second hierarchy of immunogens**

|  | MW (kDa) | pI | β-Turn | Flexibility | Antigenicity | Hydrophilicity | B cell epitope | T cell epitope | Function |
| --- | --- | --- | --- | --- | --- | --- | --- | --- | --- |
| BlC | 18 | 8.88 | 0.976 | 0.985 | 1.032 | 1.169 | 0.068 | 1.1146 | Transport |
| NanC | 27.9 | 8.42 | 1.036 | 0.997 | 1.008 | 1.712 | 0.129 | 1.3632 | Transport |
| BamC | 36.8 | 5.34 | 0.994 | 1.003 | 1.03 | 1.959 | 0.333 | 1.0988 | Outer membrane assembly |
| OmpF | 39.3 | 4.76 | 1.045 | 1 | 1.006 | 2.077 | 0.291 | 1.2222 | Transport |
| OmpG | 34.9 | 4.45 | 1.027 | 0.988 | 0.999 | 1.739 | 0.202 | 1.072 | Transport |
| OmpW | 22.9 | 6.03 | 0.994 | 0.987 | 1.016 | 1.199 | 0.045 | 0.9106 | Transport |
| PaL | 19 | 6.29 | 1.016 | 1.001 | 1.005 | 2.257 | 0.232 | 1.1468 | Unknown |
| BamA | 90.6 | 4.93 | 1.025 | 1.003 | 1.015 | 1.91 | 0.274 | 1.3746 | Outer membrane assembly |
| YedS | 43 | 4.46 | 1.072 | 1.001 | 0.993 | 2.31 | 0.42 | 1.1152 | Transport |
| BamB | 41.9 | 4.72 | 0.992 | 1.003 | 1.036 | 1.404 | 0.077 | 1.4748 | Information transfer |
| YieC | 60.7 | 5.22 | 1.018 | 1.005 | 1 | 1.985 | 0.29 | 1.1174 | Transport |
| YbiL | 81.96 | 5.55 | 1.045 | 1.011 | 1.003 | 2.347 | 0.499 | 1.3012 | Transport |
| FimD | 96.5 | 6.64 | 1.033 | 1 | 1.016 | 1.775 | 0.223 | 1.1456 | Transport |
| TolC | 53.7 | 5.46 | 0.997 | 1.004 | 1.022 | 2.068 | 0.278 | 1.4246 | Transport |
| YbhC | 46.3 | 5.66 | 1.032 | 1.003 | 1.018 | 2.007 | 0.412 | 1.2908 | Cell wall modification |

**Table S3 Physical, chemical, and immunological characteristics of the third hierarchy of immunogens**

|  | MW (kDa) | pI | β-Turn | Flexibility | Antigenicity | Hydrophilicity | B cell epitope | T cell epitope | Function |
| --- | --- | --- | --- | --- | --- | --- | --- | --- | --- |
| FepA | 82.1 | 5.39 | 1.038 | 1.009 | 0.999 | 2.122 | 0.427 | 1.1578 | Transport |
| FhuE | 81(38) | 4.75 | 1.033 | 1.006 | 1.002 | 2.001 | 0.349 | 1.1462 | Transport |
| HlpA | 15.8 | 9.69 | 0.929 | 1.009 | 1.013 | 2.331 | 0.154 | 0.8572 | Information transfer |
| MdtP | 53 | 6.85 | 0.937 | 0.985 | 1.037 | 1.208 | -0.048 | 1.1762 | Transport |
| OmpP | 35 | 5.9 | 1.04 | 1.006 | 0.997 | 1.963 | 0.321 | 1.0506 | Proteolysis |
| OmpX | 18.6 | 6.56 | 1.039 | 0.998 | 1.015 | 2.024 | 0.316 | 0.7196 | unknown |
| YraJ | 93 | 5.58 | 1.047 | 1.007 | 1.011 | 1.996 | 0.325 | 1.2076 | Transport |
| YbgQ | 90.1 | 5.03 | 1.037 | 1 | 1.013 | 1.813 | 0.25 | 1.2922 | Transport |
| YejO | 91.2 | 4.71 | 1.017 | 1.004 | 1.008 | 2.081 | 0.287 | 1.289 | Cell adhesion |
| YohG | 43 | 7.88 | 0.941 | 0.994 | 1.021 | 1.626 | 0.096 | 1.0864 | Transport |
| YqhH | 9.5 | 8.8 | 0.927 | 0.984 | 1.05 | 1.449 | -0.319 | 0.8258 | unknown |
| FadL | 48.5 | 5.09 | 1.034 | 0.999 | 1.006 | 1.679 | 0.29 | 1.1178 | Transport |
| FecA | 85 | 5.59 | 1.039 | 1.005 | 1.008 | 1.911 | 0.372 | 1.2726 | Transport |
| RzoD | 6.7 | 9.75 | 1.01 | 0.985 | 1.066 | 0.328 | 0.132 | 0.4874 | Cell wall macromole catabolic process |
| FhuA | 82.2 | 5.47 | 1.023 | 1.003 | 1.018 | 2.089 | 0.408 | 1.1334 | Transport |
| CirA | 73.9 | 5.11 | 1.041 | 1.011 | 1.008 | 2.157 | 0.399 | 1.3478 | Transport |

**Table S4 Physical, chemical, and immunological characteristics of the fourth hierarchy of immunogens**

|  | MW (kDa) | pI | β-Turn | Flexibility | Antigenicity | Hydrophilicity | B cell epitope | T cell epitope | Function |
| --- | --- | --- | --- | --- | --- | --- | --- | --- | --- |
| FlgH | 24.6 | 7.87 | 1.031 | 1.007 | 1.021 | 1.783 | 0.333 | 1.1058 | Metabolism |
| YccZ | 41 | 5.74 | 0.968 | 0.996 | 1.028 | 1.301 | 0.048 | 1.4586 | Transport |
| OstA | 89.6 | 4.94 | 1.035 | 1 | 1.012 | 1.913 | 0.295 | 1.2518 | Metabolism |
| MltB | 40.2 | 9.07 | 1.006 | 0.992 | 1.023 | 1.269 | 0.155 | 1.2248 | Metabolism |
| YcbS | 95.2 | 5.41 | 1.025 | 1.001 | 1.018 | 1.747 | 0.215 | 1.3696 | Transport |
| BamD | 27.8 | 6.16 | 0.974 | 0.988 | 1.023 | 1.592 | 0.149 | 1.2458 | Outer membrane assembly |
| HtrE | 95.5 | 4.88 | 1.044 | 1.004 | 1.017 | 1.903 | 0.252 | 1.3918 | Transport |
| OmpT | 35.7 | 5.76 | 1.06 | 1.007 | 0.992 | 2.053 | 0.361 | 1.1106 | Metabolism |

**Table S5 Physical, chemical, and immunological characteristics of the fifth hierarchy of immunogens**

|  | MW (kDa) | pI | β-Turn | Flexibility | Antigenicity | Hydrophilicity | B cell epitope | T cell epitope | Function |
| --- | --- | --- | --- | --- | --- | --- | --- | --- | --- |
| CusC | 50.3 | 6.05 | 0.96 | 0.996 | 1.03 | 1.432 | -0.008 | 1.214 | Response to copper ion |
| PldA | 33 | 5.15 | 1.019 | 0.991 | 1.017 | 1.264 | 0.096 | 1.0478 | Metabolism |
| MltA | 40.4 | 9.04 | 1.016 | 0.996 | 1.017 | 1.553 | 0.239 | 1.1872 | Metabolism |
| LpP | 8.3 | 9.3 | 0.99 | 1.008 | 1.027 | 2.333 | 0.126 | 0.8552 | Metabolism |
| OmpC | 40 | 4.58 | 1.059 | 1.004 | 1.003 | 2.187 | 0.323 | 1.2424 | Transport |
| NmpC | 40.3 | 4.64 | 1.018 | 0.996 | 1.008 | 2.039 | 0.244 | 1.16 | Transport |
| TsX | 33.6 | 5.07 | 1.058 | 0.991 | 0.999 | 1.375 | 0.148 | 1.1554 | Transport |
| YehB | 92.3 | 5.67 | 1.036 | 1.002 | 1.014 | 1.858 | 0.239 | 1.2376 | Transport |
| YiaT | 27.4 | 4.54 | 0.993 | 0.988 | 1.021 | 1.466 | 0.131 | 0.9782 | Unknown |
| YfcU | 97.5 | 4.71 | 1.04 | 0.999 | 1.015 | 2.029 | 0.362 | 1.2596 | Transport |
| WzA | 40 | 6.13 | 0.995 | 1 | 1.022 | 1.445 | 0.105 | 1.2758 | Transport |
